# Determinants of crop residue burning practice in the Terai region of Nepal

**DOI:** 10.1371/journal.pone.0253939

**Published:** 2021-07-01

**Authors:** Sugat B. Bajracharya, Arabinda Mishra, Amina Maharjan

**Affiliations:** International Centre for Integrated Mountain Development (ICIMOD), Kathmandu, Nepal; University of Delhi, INDIA

## Abstract

The open burning of agricultural crop residue is a key environmental issue facing the Hindu Kush Himalaya region, the Indo-Gangetic plain in particular. There is a varying intensity in the incidence of open agricultural burning in this region, and multiple drivers that determine why farmers in this region decide to burn their crop residues. While there have been research studies conducted for other countries in the region, research into the determinants of crop-burning in the Nepalese context is missing. Using primary data from a survey of 388 farming households across three districts of the Nepal Terai―Nawalparasi, Rupandehi and Kapilvastu―applying a recursive bivariate probit model, this study seeks to find out what drives the Nepalese farmers to burn their crop residue instead of using them in a sustainable manner and suggest policy recommendations for mitigation. Our findings show that the major determining factors that influence the farmers’ behavior in Nepal are livestock ownership, combine harvester use and awareness level of the farmers. While the effects of crop residue burning is transboundary in nature, the mitigation measures require to be region specific. Based on the findings, the study proposes raising livestock, using technology like Happy Seeders or upgrade the combine harvesters, raising awareness and changing perception of farmers, and promoting alternative uses of crop residue as viable mitigation measures.

## Introduction

The open burning of agricultural crop residue is a key environmental issue facing the Hindu Kush Himalaya (HKH) region, particularly the Indo-Gangetic plain (IGP), which straddles parts of northern and eastern India, eastern Pakistan, much of Bangladesh, and the southern plains of Nepal. Residue burning is one of the major sources of air pollution within and downwind of the HKH. Large agricultural fires detected by the moderate-resolution imaging spectroradiometer (MODIS) satellite imaging sensors suggest an increasing prevalence of open crop residue burning in Bangladesh, Bhutan, China, India, Nepal, and Pakistan [1]. In recent years, it has become the most common method of removing post-harvest residues of crops such as paddy, wheat, and maize in the region. This burning of biomass releases a range of air pollutants that contribute to the deterioration of air quality. It also releases several pollutants that affect the climate in the longer term, including the greenhouse gases carbon dioxide (CO_2_), nitrous oxide (N_2_O), and methane (CH_4_), and fine particles known as black carbon. The harmful effects of biomass burning range from adverse human health and diminished crop growth, to the degradation of natural ecosystems and physical infrastructure [2]. Negative effects are also seen in the form of regional climate change impacts as a result of the emissions of black carbon from the incomplete combustion of biomass. The warming effects as a result of black carbon and greenhouse gases are larger at high altitudes as they melt snowpacks and glaciers [3]. Thus, the impacts of burning crop residue go beyond the immediate area burnt, to a large part of the HKH region.

The growing demand for food has meant that the intensity of agro-based activities has increased tremendously, particularly in the Indo-Gangetic plain. The region has seen a large-scale commercialization of agriculture and mechanization of agricultural processes. With the introduction of modern farming methods, traditional practices are slowly being replaced. Traditionally, in an integrated crop‒livestock farming system, paddy and wheat crops were harvested manually, which left only short stalks in the field. The harvested straw was used for livestock and household purposes such as roofing, cooking, etc. However, with increasing commercialization and specialization in farming, using machines to harvest the crop is on the rise. This mechanization of harvesting leaves longer stalks in the fields, which causes problems for the next crop. Additionally, the use of crop residue is on the decline due to reduced demand following changes in livestock keeping, type of household, and in the use of cooking fuels. This has created a challenge in disposing of the large amount of agricultural residues produced [4]. To add to this challenge, a large amount of the crop residue needs to be disposed of in the short period of time between the harvesting of one crop and the planting of the next. This has led to the tendency of farmers burning the stalks in their fields itself, as it is a cheaper and more convenient way to dispose of the crop residue quickly. This burning of crop residue is one of the most significant sources of pollution in the IGP and beyond during the rice harvesting season (October‒November) and the wheat harvesting season (April‒May).

To understand the determinants of crop residue burning, it is important to delve into the residue management behavior of the farmers. Farmers tend to adopt a variety of residue management techniques that include leaving residues for mulching, burying, and re-ploughing in the field; selling it; making straw mattresses; roofing/thatching; space heating during winter; burning it as fuel for cooking; using it for animal bedding; as fodder; and outright burning [5]. Multiple residue management techniques are used by the same farmer depending on their choices. These methods can be divided into two broader categories: (a) sustainable use of the residue, and (b) burning it.

There are a number of determining factors that farmers consider in deciding whether to use the residue sustainably or not. Although there has been growing evidence regarding factors determining crop residue burning in Asia in general, research in the Nepalese context has hitherto been missing. While the effects of crop residue burning is transboundary in nature, the mitigation measures require to be region specific. In order to effectively design relevant mitigation measures, it is vital to find out the reasons why farmers do not utilize crop residues in a sustainable manner. Hence, it is the objective of this paper to identify the determinants of crop residue burning in Nepal. Subsequently, this can aid policy makers in designing mitigation measures geared towards addressing them in the Nepalese context.

## Literature review of determinants

The literature on crop residue burning in South Asia has mostly focused on emission measurements [6–11], the impact on air quality [12–14], and its adverse effects on human health and the environment [15–21]. As Nowak [22] indicated, efforts to increase the adoption of environment-friendly residue management practices need to be based on an understanding of why farmers reject them, or do not take these up in the first place. However, the review of literature shows that there have been few studies assessing the determinants of open crop residue burning in the IGP region [23–26].

A review of the studies that analyse factors underlying crop residue burning decisions and practices of farmers in the South Asian context show a diversity of methodologies used in assessment. For instance, Ahmed and Ahmad [24] assess the determinants of open crop residue burning by identifying four different residue management practices in Punjab, Pakistan, by assuming that the extent of adoption of each residue management practice can be represented by the percentage area under each practice. Gupta [23], on the other hand, assesses the determinants of open residue burning by treating the choice of harvesting method and that of residue burning as two interdependent decisions. Similarly, other studies use variations of probit models to assess determinants.

From the review of literature for the region, we find that multiple determinants affect farmers’ decisions in choosing to burn the crop residue or adopt a residue management practice. The influencing factors that have emerged from the review are ownership of livestock, use of combine harvesters, straw length, farm size, geographical location of the farm, altitude at which the farm is located, gap between two seasons, demographic characteristics, awareness about the negative effects of burning residue, access to non-agricultural incomes, and regulations geared towards alleviating the open burning of crop residue.

Ahmed and Ahmad [24], Hu [27], and Chendrashekhara et al. [26] report that livestock ownership, or number of animals in a farm, has a significantly positive bearing on farmers choosing to use the residue comprehensively. These studies show that households with a significant number of livestock tend to remove the residue over a significantly larger area. Launio et al. [28] report similar findings in the Philippines. This study shows that cow ownership is a significant variable that encourages opting for straw use and removal over burning. However, Fang et al. [29] present contrary results; they show that higher per capita animal husbandry looked to have a positive effect on the incidence of straw burning.

Similarly, the use of combine harvesters has a major influence on whether crop residues get burnt or not. The use of combine harvesters has gained in popularity in the region as a result of a shortage of labour. As these machines leave behind residue unfit for most uses, their rampant use has been associated with an increasing prevalence of burning. For instance, Gupta [23] finds that the use of combine harvesters had a substantial effect, on average, on the probability that farmers would burn their crop residue. This study showed that plots on which combine harvesters were used were 80 per cent more likely to have their residue burnt than plots using a manual form of harvesting. Yang et al. [30] attribute open field burning of cereal residues in Suqian region in Jiangsu province, China, from 2001‒2005 to the use of combine harvesters. Farooq et al. [31] find similar results in the province of Punjab in Pakistan. Similarly, Haider [25] finds that straw length positively and significantly influences the choice made regarding burning paddy residue.

Farm size is both positively and negatively associated with the incidence of burning in various geographical locations, highlighting the importance of a location-specific context. Ahmed and Ahmad [24] and Haider [25] find a positive correlation between increase in farm size and the incidence of burning of residue in Pakistan and Bangladesh respectively. As farm size tends to mirror the volume of residue produced, the increased incidence is associated with more residue burning as well. However, Hu [27] shows, to the contrary, that farmers in China with larger planting acreage tend to utilize the crop residue comprehensively.

The altitude at which the farm is located also influences the incidence of burning. Location of paddy farms at lower elevations significantly increases the likelihood of the residue being burnt in the field [25]. Fang et al. [29] report similar findings from Northeast China, where they find a greater incidence of straw burning concentrated in farms located on the lower and lowest slopes. Furthermore, the distance to the plot from the farmer’s homestead positively and significantly influences the incidence of paddy residue burning [25]. This is corroborated by Launio et al. [28] who also find that an increased distance between a farmer’s house and farm is negatively correlated to the incorporation or use of straw residue.

One characteristic that has emerged as a major factor is the gap between two seasons. Ahmed and Ahmad [24] and Haider [25] emphasise that a reduction in the turnaround time between the harvesting of rice and the sowing of wheat is associated with an increase in the burning of crop residue due to the short period of time available to prepare the fields for the next crop.

In line with the characteristics of the farm, demographic and other social factors are also found to be influential. Characteristics pertaining to income, educational levels, awareness of the problem, age, and caste tend to influence the decision-making of farmers. The findings from China by Hu [27] show that farmers who earn higher non-agricultural incomes are more reluctant to utilize the straw comprehensively. This study also finds that older and more educated farmers are less inclined to utilize crop residue. In contrast, Gupta [23] and Haider [25] don’t find any statistically significant influence of farmers’ age on decision-making regarding crop-burning. Similarly, education level of the farmers also does not seem to influence decision-making. Farmers who are aware that burning paddy residue has a negative effect on the environment, paradoxically, tend to be associated with an increase in the amount of agricultural land over which residue is burnt [24]. However, the opposite is seen to be true in the case of China [27]. Regarding caste as a factor, Ahmed and Ahmad [24] find that farmers belonging to the Rajput caste were less likely to adopt residue management methods of full utilization compared to other castes in Pakistan.

This literature review of determinants shows that there are multiple factors that influence the farmers’ decision making. These factors can have varying impacts depending on the region. It shows that it is important to consider the local context and multiple factors while trying to understand farmers’ decision-making regarding the burning of crop residues, in order to formulate better policies that would mitigate it.

## Methodology

### Ethics statement

Data were obtained through face-to-face interviews conducted in the fields and/or households of the respondents. Verbal consent was obtained from all of the respondents prior to the interview. The verbal consent was taken for two reasons: first, a large proportion of the respondents were expected to be illiterate who were not comfortable signing consent forms; second, no biological data was collected from the socioeconomic survey. The interviewers explained the process and communicated that their participation was voluntary and that the data being collected were for research purposes and uses. There is no institutional review board in Nepal to review and approve non-medical research with human subjects.

### Study area

Nepal is divided into three agroecological zones, namely the mountains, the hills and the Terai. This study was conducted in three districts of the Terai―Kapilvastu, Nawalparasi, and Rupandehi. The Terai is the grain basket of the country, and all the three districts selected are big producers of grain. Data from the National Sample Census of Agriculture, Nepal 2011‒12 [32] shows that, in all three districts under study, paddy production predominates in comparison to wheat (Table 1). As a result, the crop residue from paddy is also abundant, the management of which is a challenge.

**Table 1:** Proportion of paddy and wheat grown in the study districts (2011/12).

District	Total Agricultural Land (In Hectares)	Land area under temporary crops (In Hectares)	% Agricultural land (to total district area)	% Main paddy area (to total agricultural land)	% Wheat area (to total agricultural land)	% Main paddy area (to land area under temporary crops)	% Wheat area (to land area under temporary crops)
Nawalparasi	56033.7	50637.1	25.9	68.45	23.7	75.7	26.2
Rupandehi	71119.5	65793.4	52.29	87.20	46.6	94.3	50.4
Kapilvastu	64548.7	59906	37.14	83.99	35.1	90.5	37.9

Source: Central Bureau of Statistics (2013).

In order to obtain a fairly representative sample of farmers and taking into account the rural‒urban dynamics in the Terai, one municipality and two village development committees (VDCs) in each of the three districts were identified, with the farmers selected randomly. These were selected in areas that lie close to the border with India, south of East‒West highway, as a preliminary field visit revealed that they were major open crop burning areas. Combine harvesters have been deployed in these districts since 2000. Images taken during the first week of November 2016, and accessed from Google Earth showed active fires in these three districts. Historical images of this region also show spots of agricultural fields burned in the past.

During the 2016 cropping season, rice was planted in almost all of the agricultural land in the study sites. On the other hand, wheat was planted on around 73% of the total agricultural land due to the lack of irrigation during the wheat season (Table 2). Henceforth, we focus on paddy residues in the study areas.

**Table 2:** Area under rice and wheat in the study sites (ha) in 2016.

District	Study Sites	Total Agricultural land	Total Area under rice cultivation	Total Area under wheat cultivation
Nawalparasi	Ramgram Municipality	46.61	46.19	33.75
	Hakui VDC	40.91	40.91	29.69
	Palhi VDC	27.28	27.28	20.57
Rupandehi	Lumbini Municipality	59.32	59.26	41.39
	Bagaha VDC	65.09	63.80	54.50
	Basantapur VDC	57.62	57.38	41.01
Kapilvastu	Kapilvastu Municipality	47.46	44.21	28.75
	Dumara VDC	20.26	19.59	16.43
	Pakadi VDC	30.20	30.20	23.11

### Sampling

The sample size was determined by the use of William Cochran’s sample size formula for categorical data. The minimum sample was adjudged to be 388 households. The total number of households to be sampled in the three districts was determined by using the National Population Census of 2011, as data on the number of agricultural households in the districts was not available. The number of households to be sampled in each district was determined using the Probability Proportional to Size (PPS) method. This number was equally distributed across the three study sites (two VDCs and a municipality) within each district. The number of households sampled in each of the study sites is presented in Table 3.

**Table 3:** Sampling distribution.

District	Study Site	Number of respondents
Nawalparasi	Ramgram Municipality	43
	Hakui VDC	44
	Palhi VDC	43
Rupandehi	Lumbini Cultural Municipality	59
	Bagaha VDC	54
	Basantapur VDC	55
Kapilvastu	Kapilvastu Municipality	32
	Dumara VDC	27
	Pakadi VDC	31
Grand Total		388

### Analytical approach used

The initial scoping study showed that the mode of harvesting opted for tends to strongly influence the choice of whether to burn the residue or not. We find that about 75 per cent of farmers who use a combine harvester tend to burn the paddy residue. This is due to the difficulty in retrieving the residue after using a combine harvester, as it tends to scatter the residue all over the field. We devise the model of analysis to reflect this tendency of the farmer.

We use a recursive bivariate probit model in the analysis, which stems from the fact that the choice of harvesting method and the choice of residue burning tend to be two interdependent decisions. The fact that the farmers tend to simultaneously decide on the mode of harvesting and the method of residue disposal makes the use of this model appropriate. For instance, manual harvesting allows for an easy retrieval of crop residue, hence making it less likely that it will be burnt. On the other hand, the likelihood of the farmers burning the residue increases with the use of combine harvesters.

Hence, we model our analysis on the specifications used by Gupta [23], as follows: we assume that the decision to burn the residue, or not, by the farmers is a binary one. Moreover, we also take it that the method of residue disposal would affect the choice of harvesting as outlined by Gupta [23]. However, we do not control for the method of residue disposal in the equation on the mode of harvesting as its influence is assumed to be little. The reason for this is that there is no market for paddy residue in the study districts.

Let b = 1 mean burning of residue on a plot and b = 0 mean not burning. Similarly, c = 1 means usage of a combine harvester on a plot and c = 0 means manual harvesting.


$$b^{*}=\chi_{ibp}^{\prime }\beta_{b}+c_{p}^{\prime }\gamma +\varepsilon_{ibp}$$ Eq 1

$$c^{*}=\chi_{icp}^{\prime }\beta_{c}+\varepsilon_{icp}$$ Eq 2

$\chi_{ibp}^{\prime }$ and $\chi_{icp}^{\prime }$ are vectors of farmer and plot level attributes.$\varepsilon_{ibp}$ and $\varepsilon_{icp}$ are disturbance terms.

We seek to examine variables that have been influential in other studies in the IGP to test for their relevance and influence for analysis in Nepal. Hence, we have carefully modelled the variables keeping national characteristics in mind.

The variables and their descriptions are given in Table 4.

**Table 4:** Description of variables used in the analysis.

Variables	Description	Unit of Measurement	Variables		Variable derived from
			**Mean**	**S.D.**	
Burn straw	Method of residue disposal 1 = residue is burned 0 = otherwise	Binary response from farmer	0.59	0.49	
Combine harvester	Whether or not farmer used a combine harvester to harvest rice. 1 = Combine harvester is used 0 = otherwise	Binary response from farmer	0.73	0.44	[23]
Size of Agricultural land under rice cultivation	Agricultural land used for rice cultivation	Bigha	1.47	1.64	
Livestock	Whether the household own any livestock or not. 1 = Yes 0 = No	Binary response from farmer	0.59	0.49	[23]
Total Household Size	Total number of family members in the household.	Number	8.46	4.11	
Age of respondent		Number	40.4	14.69	
Education of respondent	Different levels of education	Number			
Labour wage (male)	Daily wage rate of labour (male)	Nepali Rupee	371.9	52.5	
Awareness of burning residue	Awareness of the negative effects of burning residue on environment. 1 = Yes 0 = No	Binary response from farmer	0.59	0.49	[24,27]
Main source of income as non-agricultural income	Whether non-agricultural income is the main source of income in the household. 1 = Yes 0 = No	Binary response from farmer	0.24	0.43	[27]

Notes: 1 bigha = 1.67 acres; S. D. = standard deviation.

## Results and discussion

**Fig 1:**
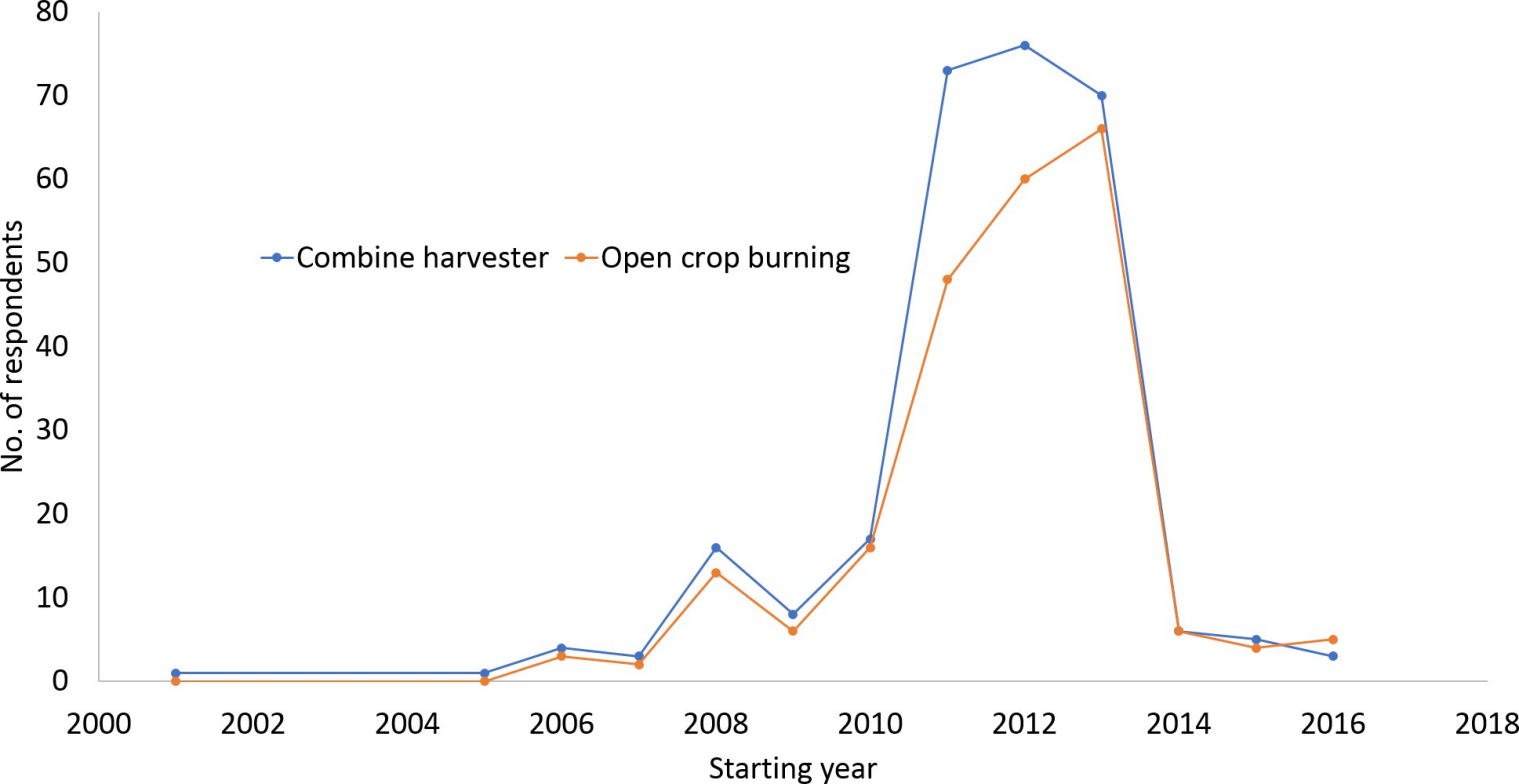
Trends in the start of combine harvester use and open residue burning.

The analysis shows that the use of a combine harvester is the major driver in determining whether the farmers in the study area burn their crop residue or not. Plots in which a combine harvester was used were found to be 54% more likely to have their crop residue burnt as compared to plots in which manual labour was deployed. This is particularly significant as a majority of farmers in the study sites use combine harvesters―over 80 per cent of farmers in Rupandehi and Kapilvastu and 68 per cent of farmers in Nawalparasi were using them. As anticipated, the increasing trend in the use of combine harvesters overlaps with the increasing trend in open residue burning (Fig 1).

Livestock ownership was seen to be another major driver in the farmer’s choice of a method of harvesting. It was observed that farmers owning livestock were 26 per cent less likely to use combine harvesters (Table 5). These farmers were more likely to harvest the crop manually and less inclined to burn the crop residue, as the residue was used in livestock feeding and bedding purposes.

**Table 5:** Marginal effect of the variables on choice of harvesting.

Variables	Probit Method (Average Marginal Effect)
Size of Agricultural land under rice cultivation	0.0184 (1.03)
Livestock	-0.260*** (-6.34)
Total Household Size	0.007 (1.20)
Age of respondent	-0.001 (-1.20)
Education of respondent	0.015 (1.38)
Labour wage (male)	0.001*** (3.05)

Notes: Dependent variable is Combine harvester. Combine harvester = 1 if the farmer used a combine harvester and 0 is otherwise. Figures in parenthesis are z-scores.

***indicates significance at 1% level

**indicates significance at 5% level

*indicates significance at 10% level.

Farmers whose main source of income was through non-farm employment were 8 per cent more likely to burn crop residue than farmers whose main source of income was from agriculture. This is seen to be the case as the opportunity costs of disposing the crop residues are higher for those who are not invested in the agricultural process.

It is also seen that the farmers who were aware of the negative effects of burning the residue were 7 per cent less likely to do so. This shows that awareness can have some positive effect on the behaviour of the farmers, in terms of their decision to burn or not to burn residue. Also, a higher household size meant that the farmer was 1.3 per cent less likely to burn the crop residue (Table 6).

**Table 6:** Marginal effect of the variables on choice of residue disposal.

Variables	Probit Method (Average Marginal Effect)
Size of Agricultural land under rice cultivation	0.014 (0.97)
Total Household Size	-0.013*** (-2.96)
Combine Harvester	0.542*** (15.75)
Awareness of burning residue	-0.072* (-1.86)
Main source of income as non-agricultural income	0.082* (1.70)

Notes: Dependent variable is Burn straw. Burn straw = 1 if the farmer burned residue and 0 is otherwise. Figures in parenthesis are z-scores.

***indicates significance at 1% level

**indicates significance at 5% level

*indicates significance at 10% level.

Relating these results to the context at hand, we can carve out the trajectory that has led to the incidence of open agricultural residue burning in Nepal. The use of combine harvesters is attributed to the lack of an available labour force, most likely due to outmigration. A lesser availability of labour results in a higher wage rate for labour, making it even harder for farmers to afford employing them. Moreover, the availability of farm hands in the form of household members also plays a part i.e. less household size is associated with more likelihood of crop residues being burned. This results in a greater use of combine harvesters, which in turn increases the likelihood of their burning crop residue. Similar results have been reported by Gupta [23].

Apart from migration, the diversified income-generating activities could be another factor leading to labour shortages in agriculture. We find that a significant proportion of households in Nawalparasi reported non-farm income as a major source of household earnings. In the same vein, our findings show that farmers who indicated non-farm incomes as their major source of earnings were more likely to burn crop residue than the farmers whose main source of income were from agriculture. These findings conform with that of Hu [27].

Farming households that owned livestock were seen as less likely to burn crop residue. This can be corroborated by findings from the field in areas east of Ramgram municipality in Nawalparasi. These areas were found to be relatively less affected by open residue burning as there were plenty of cows, bulls, and buffaloes in the area. Our findings are in line with those of Ahmed and Ahmad [24], Hu [27], Launio et al. [28], and Chendrashekhara et al. [26]. However, Fang et al. [29] report contrary results in Northeast China, where factors such as non-digestibility and poor palatability, among others, restrict the use of straw as animal feed.

Farmers who were aware of the negative effects of open crop burning on the environment were less likely to burn the crop residue. This finding is in line with Hu [27] but contradicts the findings reported by Ahmed and Ahmad [24]. Ahmed and Ahmad [24] opine that one plausible explanation for this would be that the farmers do not consider environmental impacts important enough when considering alternative residue management practices. This means that awareness alone cannot deter farmers from burning their crop residue―other characteristics of farmers such as outlook towards environmental costs and perception level may be complementary factors affecting their decisions as well. The results from Raza et al. [33] from a study in Pakistan points to this; their results indicate that farmers are more willing to adopt sustainable practices if they perceive high risks to the environment and associate it with health problems.

Demographic factors such as age, and education levels of the farmer were also analyzed to examine their influence on the farmers’ decision-making. They were not statistically significant in the choice of harvesting method or residue disposal. These results are consistent with the findings in Haider [25]. Gupta [23] also reported no influence of age of the farmer on the decision whether to burn residue or not. Contrary to this, Hu [27] shows a negative correlation between the educational level of the famer and comprehensive straw utilization; farmers with a higher educational level were less inclined to utilize the straw comprehensively. This study also revealed that older farmers tended to be less inclined to use the crop residue comprehensively.

In our study, the extent of agricultural land devoted to rice cultivation was also not seen to be statistically significant in affecting the decision-making of farmers regarding harvesting or residue disposal methods. This is consistent with the findings in Gupta [23] and Haider [25]. However, although statistically insignificant, an increase in farm size is seen to influence straw burning practice. This can also be corroborated by Ahmed and Ahmad [24] and Fang et al. [29]. However, Hu [27] reports results contrary to these findings. The influence of farm size on the decision to burn residue tends to depend on other factors such as livestock per unit area and suitability of the straw as feed for livestock. Ahmed and Ahmad [24] show that an increase in farm size is associated with fewer animals per unit area resulting in a lower use of feed by livestock. A comprehensive use of straw as a result of an increase in farm size, as reported by Hu [27], depends largely on an increase in the use of straw as livestock feed. The inability to use straw as feed could be the reason for an increase in farm size being considered less of a factor in the burning of crop residue [29].

## Conclusion and policy implications

Our findings show that the major determining factors that influence the farmers’ behavior in Nepal when it comes to crop residue burning are livestock ownership, combine harvester use and awareness level of the farmers. The demographic factors that were investigated were not seen to be statistically significant. It was also observed that the influence of all of these factors is driven by the interactions between them that affect the cognitive processes of farmers.

The existence of multiple drivers constitutes a persistent challenge in devising mitigation measures. A better understanding of farmers’ decision-making processes, as well as of the factors that drive those decisions, will, we believe, help in devising effective policies and programs to reduce the extent of residue burning. Merely regulations banning farmers from burning their crop residue is not going to work, unless the needs of the farmers are also simultaneously addressed.

By analyzing the determinants of the farmer behavior that leads to open crop burning, several cues from the findings can help to devise mitigation strategies for the relevant stakeholders and policy makers in Nepal. Following are some of the policy implications from the study:

1. Raising Livestock–
  We can largely observe from our study that there is a positive influence from raising livestock in curbing open crop burning towards its utilization as livestock feed among the Nepalese farmers in the study districts. This has been revealed through FGDs with the farmers of Nawalparasi where the incidence of burning is relatively less than other districts owing to better livestock raising/ownership in the district. FGDs also reveal that farmers in Nawalparasi and some parts of Rupandehi have started to use straw choppers with the straw being primarily used for animal feed. This could be a potential mitigation measure that can decrease the incidence of burning which has also been suggested by Das et al. [34]. Hence, encouraging an integrated farming method involving livestock would be a viable option to consider for mitigation.
2. Using technology like the Happy Seeders or upgrade the combine harvesters–
  It has been very clear from the findings that the use of combine harvester is directly linked to the incidences of burning. One of the drawbacks of the combine harvesters that leads to burning of the residue has to do with the spread-out residue. It is difficult and time-consuming to manage this and then sow seeds for the next crop (usually wheat). To make collecting the residues and sowing for the next crop convenient for the farmers, use of Happy Seeders and/or modified combine harvesters equipped with residue collector is ideal. Subsidies relating to renting/buying of these equipment would be a potential mitigation measure as they can be expensive and unaffordable for a small holder farmer. The discussion with the farmers also reveals that the farmers are willing to use the technology so long as they are economically viable.
3. Raising awareness and changing perceptions of farmers-
  Two out of five respondents were not aware of the negative effects of open burning on the environment while 36% reported that they were unaware of the health effects. Our findings in this regard point out that those who are aware of the negative effects of open burning tend to be 7% less likely to resolve to burn. Therefore, programs geared towards informing the farmers on the negative effects of open crop burning on the environment and their health is needed to inform the farmers of the consequences of burning. However, these can only be effective if the farmers are able to internalize it.
4. Promoting alternate uses of crop residue–
  According to the study, about two out of five farmers pointed to lack of alternate ways of disposing crop residues as the main reason behind burning in the field. It is therefore vital to address this issue by promoting alternate ways of disposing the crop residues like in-situ utilization as mulch, using it as raw materials for industries like brick kilns, paper production, mushroom cultivation, etc. and for alternative energy production like bio-briquette, pellets [34,35].

## References

[1] Saikawa E, Panday A, Kang S, Gautam R, Zusman E, Cong Z, et al. Air Pollution in the Hindu Kush Himalaya. In: Wester P, Mishra A, Mukherji A, Shrestha AB, editors. The Hindu Kush Himalaya Assessment: Mountains, Climate Change, Sustainability and People. Cham: Springer International Publishing; 2019. pp. 339–387. doi: 10.1007/978-3-319-92288-1_10

[2] Cassou E. Field Burning Agricultural Pollution. World Bank, 2018. Available online: https://openknowledge.worldbank.org/handle/10986/29504.

[3] Ramanathan V, Carmichael G. Global and regional climate changes due to black carbon. Nat Geosci. 2008;1: 221–227. doi: 10.1038/ngeo156

[4] Bhuvaneshwari S, Hettiarachchi H. Crop Residue Burning in India: Policy Challenges and Potential Solutions. 2019. doi: 10.3390/ijerph16050832 30866483 PMC6427124

[5] Pant K. Monetary Incentives to Reduce Open-Field Rice-Straw Burning in the Plains of Nepal. 2014.

[6] Badarinath KVS, Chand TRK, Prasad VK. Agriculture crop residue burning in the Indo-Gangetic Plains–A study using IRS-P6 AWiFS satellite data. Curr Sci. 2006;91: 1085–1089. Available: http://www.jstor.org/stable/24093988.

[7] Badarinath KVS, Kumar Kharol S, Rani Sharma A. Long-range transport of aerosols from agriculture crop residue burning in Indo-Gangetic Plains—A study using LIDAR, ground measurements and satellite data. J Atmos Solar-Terrestrial Phys. 2009;71: 112–120. doi: 10.1016/J.JASTP.2008.09.035

[8] Bray CD, Battye WH, Aneja VP. The role of biomass burning agricultural emissions in the Indo-Gangetic Plains on the air quality in New Delhi, India. Atmos Environ. 2019;218: 116983. doi: 10.1016/J.ATMOSENV.2019.116983

[9] Mishra AK, Shibata T. Synergistic analyses of optical and microphysical properties of agricultural crop residue burning aerosols over the Indo-Gangetic Basin (IGB). Atmos Environ. 2012;57: 205–218. doi: 10.1016/J.ATMOSENV.2012.04.025

[10] Rajput P, Sarin M, Sharma D, Singh D. Characteristics and emission budget of carbonaceous species from post-harvest agricultural-waste burning in source region of the Indo-Gangetic Plain. Tellus B Chem Phys Meteorol. 2014;66: 21026. doi: 10.3402/tellusb.v66.21026

[11] Ravindra K, Singh T, Mor S. Emissions of air pollutants from primary crop residue burning in India and their mitigation strategies for cleaner emissions. J Clean Prod. 2019;208: 261–273. 10.1016/j.jclepro.2018.10.031.

[12] Jethva H, Torres O, Field RD, Lyapustin A, Gautam R, Kayetha V. Connecting Crop Productivity, Residue Fires, and Air Quality over Northern India. 2019; 1–11. 10.1038/s41598-019-52799-x PMC6851147 31719586

[13] Mittal SK, Singh N, Agarwal R, Awasthi A, Gupta PK. Ambient air quality during wheat and rice crop stubble burning episodes in Patiala. Atmos Environ. 2009;43: 238–244. doi: 10.1016/J.ATMOSENV.2008.09.068

[14] Singh RP, Kaskaoutis DG. Crop Residue Burning: A Threat to South Asian Air Quality. Eos, Trans Am Geophys Union. 2014;95: 333–334. 10.1002/2014EO370001.

[15] Adler T. Respiratory health. Measuring the health effects of crop burning. Environ Health Perspect. 2010;118: A475–A475. doi: 10.1289/ehp.118-a475 21465742 PMC2974718

[16] Agarwal R, Awasthi A, Mittal S, Singh N, Gupta PK. Effects of air pollution on respiratory parameters during the wheat-residue burning in Patiala. J Med Eng Technol. 2010;34: 23–28. doi: 10.3109/03091900903261258 19824783

[17] Awasthi A, Singh N, Mittal S, Gupta PK, Agarwal R. Effects of agriculture crop residue burning on children and young on PFTs in North West India. Sci Total Environ. 2010;408: 4440–4445. doi: 10.1016/j.scitotenv.2010.06.040 20637491

[18] Gupta S. Agriculture Crop Residue Burning and Its Consequences on Respiration Health of School-Going Children. Glob Pediatr Heal. 2019;6: 2333794X19874679. doi: 10.1177/2333794X19874679 31523702 PMC6734611

[19] Kumar P, Kumar S, Joshi L. Socioeconomic and Environmental Implications of Agricultural Residue Burning: A Case Study of Punjab, India. Springer Nature PP—Cham; 2015. doi: 10.1007/978-81-322-2014-5

[20] Mathur R, Srivastava V. Crop Residue Burning: Effects on Environment: Challenges, Technologies and Solutions. 2019. pp. 127–140. doi: 10.1007/978-981-13-3272-2_9

[21] Sarkar S, Singh RP, Chauhan A. Increasing health threat to greater parts of India due to crop residue burning. Lancet Planet Heal. 2018;2: e327–e328. doi: 10.1016/S2542-5196(18)30166-9 30082044

[22] Nowak P. Why farmers adopt production technology. J soil water Conserv. 1992;47.

[23] Gupta R. Causes of Emissions from Agricultural Residue Burning in North-West India: Evaluation of a Technology Policy Response; 2012.

[24] Ahmed T, Ahmad B. Why Do Farmers Burn Rice Residue? Examining Farmers’ Choices in Punjab, Pakistan. eSocialSciences; 2013.

[25] Haider MZ. Options and Determinants of Rice Residue Management Practices in the South-West Region of Bangladesh; 2012.

[26] Chendrashekhara S, G B L, Patila S, Huchaiah L. Factors Influencing the Adoption of Paddy Straw Management Practices by Farmers of Karnataka (India). Curr Agric Res J. 2018;6: 225–232. doi: 10.12944/CARJ.6.2.13

[27] Hu Z. A Research on the Affecting Factors of Farmers’ Comprehensive Utilization of Straw in China. IOP Conf Ser Earth Environ Sci. 2018;170: 22134. doi: 10.1088/1755-1315/170/2/022134

[28] Launio CC, Asis C, Manalili R, Javier E. Economic Analysis of Rice Straw Management Alternatives and Understanding Farmers’ Choices. 2015. pp. 93–111. doi: 10.1007/978-981-287-393-4_5

[29] Fang Y, Xu K, Guo X, Hong Y. Identifying determinants of straw open field burning in northeast China: Toward greening agriculture base in newly industrializing countries. J Rural Stud. 2020;74: 111–123. doi: 10.1016/J.JRURSTUD.2019.12.013

[30] Yang S, He H, Lu S, Chen D, Zhu J. Quantification of crop residue burning in the field and its influence on ambient air quality in Suqian, China. 2008;42: 1961–1969. doi: 10.1016/j.atmosenv.2007.12.007

[31] Farooq U, Sharif M, Erenstein O. Adoption and Impacts of Zero-Tillage in the Rice-Wheat Zone of Irrigated Punjab, Pakistan. CIMMYT: International Maize and Wheat Improvement Center; 2007 Dec. Available: https://econpapers.repec.org/RePEc:ags:cimmis:56095.

[32] Central Bureau of Statistics. National Sample Census of Agriculture Nepal 2011‒12. Kathmandu: National Planning Commission Secretariat, Government of Nepal. 2013.

[33] Raza MH, Abid M, Yan T, Ali Naqvi SA, Akhtar S, Faisal M. Understanding farmers’ intentions to adopt sustainable crop residue management practices: A structural equation modeling approach. J Clean Prod. 2019;227: 613–623. 10.1016/j.jclepro.2019.04.244.

[34] Das B, Bhave P V, Praveen S, Shakya K, Maharjan B, Byanju RM. A model-ready emission inventory for crop residue open burning in the context of Nepal *. Environ Pollut. 2020;266: 115069. doi: 10.1016/j.envpol.2020.115069 32763722

[35] Kafle S, Parajuli R, Euh SH, Oh KC, Choi YS, Adhikari K, et al. Potential biomass supply for agro-pellet production from agricultural crop residue in Nepal. Energy Sources, Part A Recover Util Environ Eff. 2016;38: 149–153. doi: 10.1080/15567036.2015.1043474

